# Optimizing Contact Precautions to Curb the Spread of Antibiotic-resistant Bacteria in Hospitals: A Multicenter Cohort Study to Identify Patient Characteristics and Healthcare Personnel Interactions Associated With Transmission of Methicillin-resistant *Staphylococcus aureus*

**DOI:** 10.1093/cid/ciz621

**Published:** 2019-09-13

**Authors:** Lyndsay M O’Hara, David P Calfee, Loren G Miller, Lisa Pineles, Laurence S Magder, J Kristie Johnson, Daniel J Morgan, Anthony D Harris

**Affiliations:** 1 Department of Epidemiology and Public Health, University of Maryland School of Medicine, Baltimore; 2 Division of Infectious Diseases, Weill Cornell Medicine, New York, New York; 3 LA BioMed at Harbor–University of California–Los Angeles Medical Center, Torrance; 4 Department of Pathology, University of Maryland School of Medicine, Baltimore

**Keywords:** antibiotic-resistant bacteria, MRSA, infection prevention, contact precautions

## Abstract

**Background:**

Healthcare personnel (HCP) acquire antibiotic-resistant bacteria on their gloves and gowns when caring for intensive care unit (ICU) patients. Yet, contact precautions for patients with methicillin-resistant *Staphylococcus aureus* (MRSA) remains controversial despite existing guidelines. We sought to understand which patients are more likely to transfer MRSA to HCP and to identify which HCP interactions are more likely to lead to glove or gown contamination.

**Methods:**

This was a prospective, multicenter cohort study of cultured HCP gloves and gowns for MRSA. Samples were obtained from patients’ anterior nares, perianal area, and skin of the chest and arm to assess bacterial burden.

**Results:**

Among 402 MRSA-colonized patients with 3982 interactions, we found that HCP gloves and gowns were contaminated with MRSA 14.3% and 5.9% of the time, respectively. Contamination of either gloves or gowns occurred in 16.2% of interactions. Contamination was highest among occupational/physical therapists (odds ratio [OR], 6.96; 95% confidence interval [CI], 3.51, 13.79), respiratory therapists (OR, 5.34; 95% CI, 3.04, 9.39), and when any HCP touched the patient (OR, 2.59; 95% CI, 1.04, 6.51). Touching the endotracheal tube (OR, 1.75; 95% CI, 1.38, 2.19), bedding (OR, 1.43; 95% CI, 1.20, 1.70), and bathing (OR, 1.32; 95% CI, 1.01, 1.75) increased the odds of contamination. We found an association between increasing bacterial burden on the patient and HCP glove or gown contamination.

**Conclusions:**

Gloves and gowns are frequently contaminated with MRSA in the ICU. Hospitals may consider using fewer precautions for low-risk interactions and more for high-risk interactions and personnel.

## Teaser

Sixteen percent of gloves or gowns were contaminated with MRSA when HCP were caring for intensive care unit patients with methicillin-resistant *Staphylococcus aureus* (MRSA). Being an occupational, physical, or respiratory therapist or touching patients increases the odds of MRSA contamination. Patient MRSA bacterial burden is associated with increased contamination.

Methicillin-resistant *Staphylococcus aureus* (MRSA) infections are responsible for approximately 100 000 US hospitalizations each year, with high patient morbidity and mortality [1–3]. Healthcare personnel (HCP) acquire antibiotic-resistant bacteria on their gloves and gowns when caring for patients with antibiotic-resistant bacteria, with acquisition rates of MRSA at 17%–20% [4, 5]. While these previous MRSA investigations examined risk factors for transmission, they were limited by small numbers of patients and did not examine the relationship between nasal MRSA burden and MRSA transmission. Furthermore, what is not known is whether certain patients are more likely to transmit antibiotic-resistant bacteria to HCP and therefore to subsequent patients and, if so, what characteristics identify these patients.

The Centers for Disease Control and Prevention (CDC) continues to recommend placing patients colonized or infected with MRSA in private rooms and on contact precautions in inpatient acute care settings [6, 7], yet controversy exists surrounding the pros and cons of contact precautions for patients with MRSA [8–10]. As healthcare institutions across the country struggle with the decision of if and how they should use contact precautions, more evidence is needed to guide the optimal use of contact precautions and other infection control interventions as we work to curb the spread of multidrug-resistant bacteria.

The aim of this multicenter cohort study was to understand which patient characteristics, patient care, and environmental interactions and which HCP characteristics are more likely to lead to MRSA transmission from patient to HCP. More specifically, we aim to evaluate the association between the type of HCP, contact with the patient or environment, and specific activities performed in the room and the contamination of HCP gloves or gowns with MRSA. We also assess the association between the patient’s bacterial burden and glove or gown contamination.

## METHODS

### Study Design and Participants

We performed a prospective, multicenter cohort study to determine which HCP types and patient care interactions are risk factors for MRSA transmission to HCP gloves or gowns, a surrogate for potential transmission to other patients in the intensive care unit (ICU). Between January 2016 and August 2018, MRSA-colonized patients currently admitted in any ICU were enrolled at 2 hospitals in Baltimore, Maryland, 1 in Torrance, California, and 1 in New York, New York. Between May 2017 and August 2018, we collected patient samples to assess bacterial burden. Eligible patients had a surveillance or clinical culture positive for MRSA within 7 days of enrollment. The institutional review board at each facility granted approval for waived consent of participants.

### Data Collection

We observed and cultured the gloves and gowns of 10 HCP per patient. All patient care interactions were recorded by research staff on a standardized data collection form (Supplementary Materials). Interactions were categorized into 2 domains: interactions with the patient domain or interactions with the environmental domain. These patient care interactions were selected based on prior literature that suggests that these interactions were associated with increased transmission of several types of antibiotic-resistant bacteria and previous work on MRSA and vancomycin-resistant enterococci (VRE) [5, 11–14].

Study researchers conferred with the nursing staff to obtain patient clinical characteristics at the time of observation. Following patient care, but prior to doffing, the gloves and gown of each HCP observed were sampled with a BBL dual-tipped CultureSwab (Becton Dickinson, Sparks, MD). Using a twirling motion, the swab was rubbed along each finger and the palm of both gloved hands. HCP gowns were sampled twice along both forearms and then in a “W” pattern along the beltline as done in previous studies [4, 5, 13, 14]. To quantify MRSA burden on a subset of patients, we used ESwabs (Copan Diagnostics, Murrieta, CA) to swab the patient’s anterior nares and skin in the perianal region. Finally, we swabbed the skin on the patient’s chest and arm (antecubital fossa) using a 10 × 10 cm [2] sterile stencil.

### Laboratory Procedures

For the HCP gloves and gowns, each swab was enriched overnight in Tryptic Soy Broth with 6.5% salt (Becton Dickinson, Sparks, MD) and plated to a CHROMagar Staph aureus (Becton Dickinson, Sparks, MD). All rose/mauve colonies were confirmed as *S. aureus* by Staphauruex latex agglutination and confirmed by susceptibility testing as MRSA following the Clinical and Laboratory Standards Institute guidelines [15].

For patient samples, each ESwab was vortexed in transport media, and then 5–10 serial 1:10 dilutions in Butterfield’s buffer with a final volume of 1 mL was prepared. Next, 100 µL of each dilution was spread onto CHROMagar MRSA (Becton Dickinson, Sparks, MD) in triplicate and incubated overnight at 35°C. All rose/mauve colonies were counted, and the 3 plates were averaged for a count of colony-forming units per milliliter (CFU/mL).

### Statistical Analyses

Frequencies, proportions, means, and standard deviations were calculated to describe clinical characteristics. Patient bacterial burden (x + 1) was log transformed and expressed in log_10_ CFU/mL or colony-forming units per centimeter squared (CFU/cm^2^). We estimated the following associations with HCP glove or gown contamination: HCP type, patient or environmental domain, specific patient care interactions, and patient bacterial burden. Risk factors significant at α ≤ 0.05 in the previous analyses were considered candidate predictors for the multivariable model. All models were built using logistic regression models fit by generalized estimating equations with an exchangeable correlation matrix to take into account within-patient correlation and conducted in a stepwise fashion where the model with the lowest quasi-information criterion was chosen as the final multivariable model. Potential confounders were selected a priori for all models and included HCP type and time spent in the room. All analyses were conducted using SAS version 9.4 (SAS Institute, Cary, NC).

## RESULTS

### Patient and HCP Risk Factors

Our final cohort consisted of 402 patients with MRSA. We observed 3982 healthcare worker–patient interactions. More than half of the patients enrolled had a wound (n = 238, 59%), an endotracheal tube (n = 231, 57%), an indwelling urinary catheter (n = 228, 57%), a central venous catheter (n = 226, 56%), or a nasogastric tube (n = 223, 55%) at the time of observation. Most patients (N = 357, 89%) had at least 1 of these patient characteristics or others shown in Table 1 at the time of observation. Among all HCP–patient interactions, 570/3982 (14.3%) led to contamination of HCP gloves and 233/3980 (5.9%) led to contamination of HCP gowns. Contamination of either gloves or gowns occurred 16.2% of the time

**Table 1.** Description of Enrolled Patients With Methicillin-resistant *Staphylococcus aureus* (N = 402)

Study Site	n (%)
Maryland, hospital A	263 (65.4)
Maryland, hospital B	42 (10.4)
California	53 (13.2)
New York	44 (10.9)
Clinical characteristic	n (%)
Wound	238 (59.2)
Endotracheal tube	231 (57.5)
Indwelling urinary catheter	228 (56.7)
Central venous catheter	226 (56.2)
Nasogastric tube	223 (55.5)
Diarrhea	95 (23.6)
Surgical drain	76 (18.9)
Rectal tube	65 (16.2)
Chest tube	31 (7.7)

During our observations, HCP spent a median of 8 minutes (interquartile range [IQR], 9) in the patient’s room. The odds of contamination of gloves or gown differed by HCP type (Table 2). Occupational and physical therapists had the highest odds of glove or gown contamination (odds ratio [OR], 6.96; 95% confidence interval [CI], 3.51, 13.79), followed by respiratory therapists (OR, 5.34; 95% CI, 3.04, 9.39), nurses (OR, 3.09; 95% CI, 1.84, 5.19), patient care technicians (OR, 2.02; 95% CI, 1.09, 3.74), doctors/nurse practitioners (OR, 1.83; 95% CI, 1.04, 3.25), and environmental services employees (OR, 0.98; 95% CI, 0.46, 2.09) when compared with HCP in the “other” category (eg, social workers, nutritionists),

**Table 2.** Adjusted Association Between Healthcare Personnel Type and Contamination of Gloves or Gowns With Methicillin-resistant *Staphylococcus aureus*

Type of Healthcare Personnel (N = 3982)	Number of Gloves or Gowns With MRSA/Number of Observations (% Gloves or Gowns With MRSA)	Odds Ratio (95% Confidence Interval)
Occupational/physical therapist	27/83 (32.5)	6.96 (3.51, 13.79)
Respiratory therapist	87/322 (27.0)	5.34 (3.04, 9.39)
Nurse	404/2292 (17.6)	3.09 (1.84, 5.19)
Patient care technician	36/293 (12.3)	2.02 (1.09, 3.74)
Medical doctor/nurse practitioner	61/541 (11.3)	1.83 (1.04, 3.25)
Environmental services	13/204 (6.4)	0.98 (0.46, 2.09)
Other^a^	16/247 (6.5)	Ref

Adjusted for time spent in the patient’s room.

^a^Includes social workers, nutritionists, researchers, and similar personnel.

Abbreviation: MRSA, methicillin-resistant *Staphylococcus aureus.*

On average, HCP touched 2 items in the patient domain and 2 environmental items each time they went into the patient’s room. The odds of their gloves or gowns being contaminated with MRSA increased with the number of different items touched in the patient domain (OR, 1.20; 95% CI, 1.11, 1.29 per item touched) but not with the number of different items touched in the environment (OR, 1.01; 95% CI, 0.94, 1.08 per item touched). The odds of glove or gown contamination also increased when the HCP touched the patient domain (observations where they interacted with the patient only or with both the patient and the environment) (OR, 2.59; 95% CI, 1.04, 6.51) when compared with touching nothing at all (Table 3). Touching only the environment was not associated with glove or gown contamination (OR, 1.13; 95% CI, 0.43, 3.00) when compared with touching nothing in the room. Table 3 also shows that the crude proportion of gowns contaminated with MRSA was higher when the HCP touched the patient (6.8%) than when they touched the environment only (1.5%).

**Table 3.** Adjusted Association Between Patient and Environmental Domains and Contamination of Gloves or Gowns With Methicillin-resistant *Staphylococcus aureus*

Domain Touched (N = 3982)	Number of Gloves or Gowns With MRSA/Number of Observations (% Gloves or Gowns With MRSA)	Odds Ratio (95% Confidence Interval)
Contamination of gloves or gowns		
Any patient contact^a^	594/3274 (18.1)	2.59 (1.04, 6.51)
Environment only	45/620 (7.3)	1.13 (0.43, 3.00)
Nothing	5/88 (5.7)	Ref
Contamination of gowns only		
Any patient contact ^a^	222/3273 (6.8)	1.90 (0.45, 8.00)
Environment only	9/619 (1.5)	0.66 (0.13, 3.19)
Nothing	2/88 (2.3)	Ref

Adjusted for healthcare personnel (HCP) type and time spent in the patient’s room.

^a^Includes interactions where the HCP touched the patient only as well as interactions where the HCP touched both the patient and the environment in the same interaction.

Abbreviation: MRSA, methicillin-resistant *Staphylococcus aureus*

**Figure 1.**
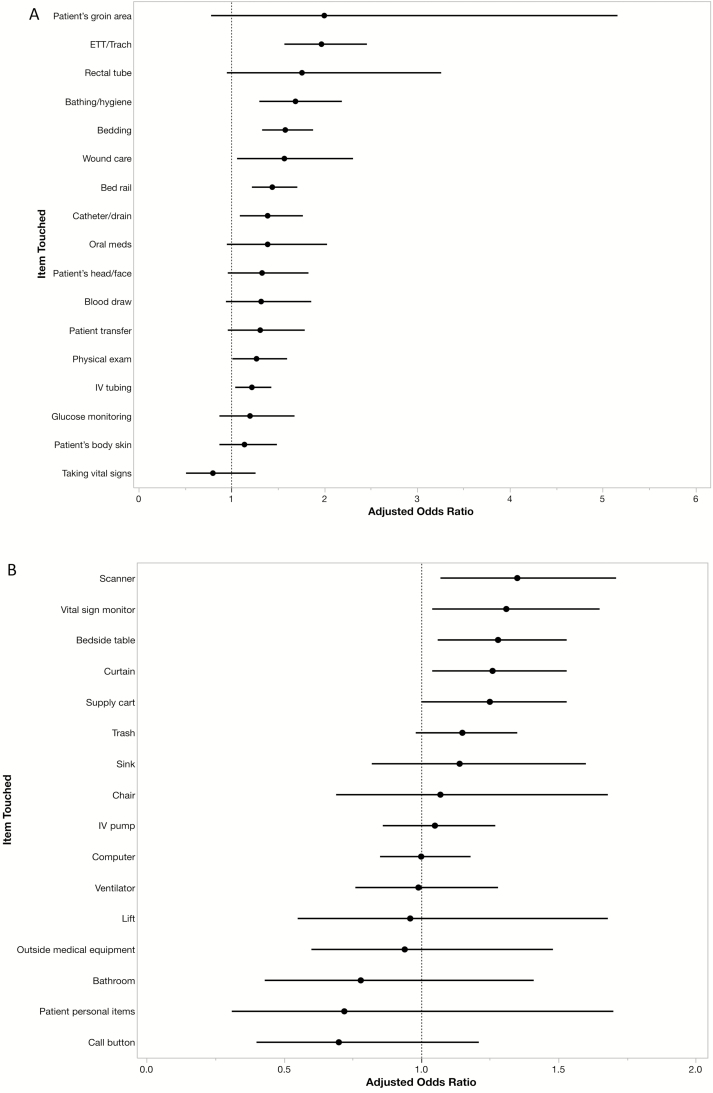
Adjusted odds ratios and 95% confidence intervals of HCP glove or gown contamination for each individual patient care activity. (*A*) Patient domain, adjusted for HCP type. (*B*) Environmental domain, adjusted for HCP type. Abbreviations: ETT, endotracheal tube; HCP, healthcare personnel; IV, intravenous.

Figure 1A and B show the adjusted ORs and 95% CIs for the outcome of HCP glove or gown contamination for each patient care interaction. In the patient domain (Figure 1A), touching the endotracheal tube (OR, 1.97; 95% CI, 1.57, 2.46), bathing the patient (OR, 1.69; 95% CI, 1.30, 2.19), touching the bedding (OR, 1.58; 95% CI, 1.33, 1.88), performing wound care (OR, 1.57; 95% CI, 1.06, 2.31), touching the bedrail (OR, 1.44; 95% CI, 1.22, 1.71), touching the catheter or drain (OR, 1.39; 95% CI, 1.09, 1.77), performing a physical exam (OR, 1.27; 95% CI, 1.01, 1.60), and touching the intravenous tubing (OR, 1.22; 95% CI, 1.04, 1.43) were associated with increased odds of transmission when compared with not touching these individual items and when adjusted for HCP type. In the environmental domain (Figure 1B), touching the barcode scanner (OR, 1.35; 95% CI, 1.07, 1.71), vital sign monitor (OR, 1.31; 95% CI, 1.04, 1.65), bedside table (OR, 1.28; 95% CI, 1.06, 1.53), and the curtain (OR, 1.26; 95% CI, 1.04, 1.53) were all associated with increased odds of transmission when compared with not touching these items and adjusted for HCP type. In a multivariable model, we found that touching the endotracheal tube (OR, 1.75; 95% CI, 1.38, 2.19), touching the bedding (OR, 1.43; 95% CI, 1.20, 1.70), and bathing the patient or assisting them with personal hygiene activities (OR, 1.32; 95% CI, 1.01, 1.75) were independent predictors for glove or gown contamination when adjusted for HCP type and total time spent in the patient’s room.

### MRSA Bacterial Burden

We obtained anterior nares, perianal, and chest or arm skin cultures from 101 patients with MRSA. The median bacterial burden in the nares was 445 CFU/mL (IQR, 775 000) and 0 CFU/mL (IQR, 1) in the perianal region. The median bacterial burden for the arm skin site was 0 CFU/mL (IQR, 0) and 0 CFU/mL (IQR, 0.75) on the chest skin site. In the nares, 59 patients (58%) had a bacterial burden >0 CFU/mL, 23 (24%) had a bacterial burden >0 CFU/mL in the perianal site, 15 (15%) on the arm site, and 25 patients (25%) had an MRSA bacterial burden on the chest site that was >0 CFU/mL. We found an association between increasing bacterial burden and HCP glove or gown contamination for 3 of 4 body sites sampled (nares: OR, 1.10; 95% CI 1.04, 1.16; the perianal: OR, 1.28; 95% CI, 1.15, 1.43; and chest: OR, 1.25; 95% CI, 1.12, 1.40; Table 4). There was no association between bacterial burden on the arm and contamination. When we compared patients with any detectable MRSA in their nares (≥1 CFU/mL or 1 CFU/cm^2^) with patients who had no detectable MRSA in their nares, we found that there was a 192% increase in HCP glove and gown contamination (OR, 2.92; 95% CI, 1.93, 4.41). Similarly, contamination increased by 57% for patients with any detectable MRSA on the perianal sample (OR, 1.57; 95% CI, 1.09, 2.25) and by 169% for patients with any MRSA on the chest (OR, 2.69; 95% CI, 1.87, 3.86).

**Table 4.** Association Between Methicillin-resistant *Staphylococcus aureus* (MRSA) Bacterial Burden and Contamination of Gloves or Gowns With MRSA by Body Site

Body Site Sampled	Odds Ratio (95% Confidence Interval)
Anterior nares (log_10_ CFU/mL)	1.10 (1.04, 1.16)
Perianal (log_10_ CFU/mL)	1.28 (1.15, 1.43)
Arm skin (log_10_ CFU/cm^2^)	1.10 (0.95, 1.28)
Chest skin (log_10_ CFU/cm^2^)	1.25 (1.12, 1.40)

Abbreviation: CFU, colony-forming unit.

## DISCUSSION

This multicenter study demonstrates that contamination of HCP gloves and gowns with MRSA occurs frequently when patients are cared for in the ICU. In our sample, we found that HCP gloves and gowns became contaminated 16% of the time. The surrogate outcome of glove and gown contamination is an important step toward understanding patient MRSA transmission. Previous studies have demonstrated extremely low colonization frequency of healthcare providers prior to donning gloves and gowns [16]. Thus, our surrogate outcome represents healthcare worker contamination with MRSA acquired from the patient or the patient’s environment during the healthcare provider interaction in nearly all instances. Our surrogate outcome is important because if the healthcare provider is not wearing gloves and a gown or performs substandard hand hygiene on room exit, MRSA represents the bacteria that then could be “transported” to the next patient that the healthcare worker visits. While hand hygiene could prevent some transmission, hand hygiene adherence is far from 100% in most institutions, difficult to improve, and cannot eradicate MRSA on HCP clothing [17]. Several investigations have demonstrated efficient transfer of bacteria from nongloved hands and healthcare provider clothing to patients and environmental surfaces [4, 8, 18]. Furthermore, MRSA has been known to persist on a variety of environmental surfaces for weeks [19].

Our data suggest that the likelihood of contamination is greater among certain types of HCP. For example, occupational and physical therapists had an almost 7-fold odds of MRSA acquisition when compared with HCP such as social workers or nutritionists. In prior investigations, we also found that occupational and physical therapists had the highest odds of acquiring VRE [11], suggesting that HCP who have direct and sustained contact with patients are at greatest risk of antibiotic-resistant bacterial contamination. Since most occupational and physical therapists, respiratory therapists, and patient care technicians provide care to many different patients on a given unit, it is especially critical that these individuals be well-versed in the proper use of personal protective equipment and hand hygiene to ensure that they are not unintentionally transferring antibiotic-resistant bacteria from room to room.

Our findings are consistent with those of several other investigations [11, 13] where direct patient contact was identified as a risk factor for transmission of antibiotic-resistant bacteria. The results presented here suggest that gloves or gowns were more than 2 times as likely to be contaminated when the HCP touched the patient compared with touching nothing in the room. Touching only the environment was not associated with glove or gown contamination. Notably, 7% of gloves or gowns and only 1.5% of gowns alone were contaminated with MRSA when the HCP touched only the environment. The lower transmission rate for environmental-only contact is important when hospitals consider removing contact precautions.

There was a 10% increase in HCP glove and gown contamination for each log_10_ increase in nose bacterial burden, a 28% increase for each log_10_ increase in perianal bacterial burden, and a 25% increase for each log_10_ increase in bacterial burden on the patient’s chest. The MRSA burden on the patient’s arm was not associated with glove or gown contamination. We also observed that bacterial burden on all body sites sampled for most patients was quite low and that any detectable level of MRSA on these 3 body sites increased the odds of transmission. These findings provide additional evidence to support interventions that aim to decrease bacterial burden, such as chlorhexidine gluconate bathing [20, 21] and nasal decolonization with mupirocin [22].

Despite being the largest study of this type to date, our study has limitations. We did not culture items in the patient’s environment and did not collect any information regarding quality and timing of room cleaning. Second, we did not culture the entire surface of the HCP gloves and gowns and thus might have underestimated the percent of gowns that became contaminated. However, we did use a standardized technique thought to maximize recovery of MRSA without interfering with patient care. We also acknowledge that high rates of glove and gown contamination do not necessarily lead to high rates of subsequent patient transmission. A large portion of our sample (65%) was enrolled at 1 hospital in Maryland and therefore findings might be most appropriately generalized to similar large, tertiary care centers. Finally, all patients enrolled in this study were in the ICU at the time of observation. Therefore, our findings may not be generalizable to patients in non-ICU areas. Further research is necessary to explore risk factors for MRSA transmission in those settings.

Our results suggest that the risk of MRSA transmission differs by HCP type and type of patient care interaction. Therefore, instead of removing all use of contact precautions or choosing to keep them for all patients with antibiotic-resistant bacteria, we suggest that hospitals consider selectively mandating precautions for high-risk activities and HCP. This would be a departure from existing CDC guidelines. However, targeted evidence-based implementation of contact precautions for patients with MRSA is a potential strategy to mitigate complete removal of contact precautions for this population as some hospitals are choosing to do. For example, our data suggest that if HCP enter a patient’s room and plan to interact with only the environment (eg, use the computer), gloves and a gown may not be necessary for a hospital looking to reduce costs by using fewer contact precautions. However, hospitals may want to mandate occupational therapists, physical therapists, and respiratory therapists to always gown and glove when caring for patients with MRSA to prevent transmission. Similarly, if HCP will be touching the endotracheal tube or the bedding or bathing the patient, it may make sense to mandate contact precautions. We acknowledge that the implementation of a risk-stratified approach to contact precautions as proposed here would need to be implemented carefully as it could be more complicated than current practice. It would be important to assess adherence to recommendations and acceptability by staff. However, we believe that this novel approach is feasible since most HCP plan their patient care activities before they enter the patient’s room. Further, this approach is responsive to the view that contact precautions are overused yet avoids total removal of contact precautions for all MRSA patients.

As hospitals continue to weigh the advantages and disadvantages of using contact precautions for antibiotic-resistant bacteria such as MRSA, we believe our findings should prompt future interventions to implement and test a more targeted, evidence-based contact precautions strategy.

## Supplementary Data

Supplementary materials are available at *Clinical Infectious Diseases* online. Consisting of data provided by the authors to benefit the reader, the posted materials are not copyedited and are the sole responsibility of the authors, so questions or comments should be addressed to the corresponding author.

ciz621_suppl_Supplementary_Information

## Notes

***Acknowledgments***. The authors thank LiCheng Zhao and Stephanie Hitchcock for specimen processing. They also thank Georgia Papaminas, Shirley Goodman, Evelyn Flores, Bryn Launer, Erin Rieger, and Barbara Kuklinksa for help with data collection and specimen shipping.

***Disclaimer.*** This work represents the findings of the authors and not necessarily the views of the US Centers for Disease Control and Prevention (CDC) or the National Institutes of Health (NIH).

***Financial support.*** This work was supported by the CDC Prevention Epicenter Program (U43CK000450-01 to A. D. H.) and the NIH National Institute of Allergy and Infectious Diseases (R01 AI121146-01 to A. D. H.). L. M. O. acknowledges support from the Canadian Institutes for Health Research Banting Postdoctoral Fellowship Program.

***Supplement sponsorship.* This article appears as part of the supplement “Personal Protective Equipment for Preventing Contact Transmission of Pathogens: Innovations from CDC’s Prevention Epicenters Program,” sponsored by the CDC’s Prevention Epicenters Program.**

***Potential conflicts of interest.*** D. J. M. reports grants from the CDC, NIH, Agency for Healthcare Research and Quality and Veterans Affairs Health Services Research & Development; payments from the Infectious Diseases Society of America and Society for Healthcare Epidemiology of America for expenses to organize or present at national meetings; and honoraria for textbook/journal editing from Springer Nature Inc outside the submitted work. L. G. M. reports grants from Genentech, Merck, AtoxBio, Tetraphase, Parateck, and Cepheid. All other authors report no potential conflicts. All authors have submitted the ICMJE Form for Disclosure of Potential Conflicts of Interest. Conflicts that the editors consider relevant to the content of the manuscript have been disclosed.
