# Supplementary material for: Optimizing Contact Precautions to Curb the Spread of Antibiotic-resistant Bacteria in Hospitals: A Multicenter Cohort Study to Identify Patient Characteristics and Healthcare Personnel Interactions Associated With Transmission of Methicillin-resistant Staphylococcus aureus
Source: Clin Infect Dis. 2019 Sep 13;69(Suppl 3):S171–7. doi: 10.1093/cid/ciz621 (PMC6761365; doi:10.1093/cid/ciz621)
Supplement: ciz621_suppl_Supplementary_Information [file ciz621_suppl_supplementary_information.docx]

**Appendix A.** Data collection forms

**PATIENT INFORMATION FORM**

| REPID (MD only): |  | Study Number: |  | Enrollment Date: |  |
| --- | --- | --- | --- | --- | --- |

**Location information**

| Midtown | UMMC | Unit/Ward: _________________________ Room: _________________________ | |
| --- | --- | --- | --- |
| NYP/WCMC | Harbor-UCLA | Pittsburgh |  |

**Culture information**

| MRSA  CRE | Culture date: ________________________________________  Final result date: ____________________________________  Accession number: __________________________________  If CRE, Organism: ___________________________________ | Culture source:  Nasal surveillance swab  Perirectal surveillance swab  Wound  Blood  Sputum  Urine  Other (specify): _______________________ |
| --- | --- | --- |

| **Date** | **Artificial airway (trach/ET tube)** | **Wound** | **Foley** | **Intravascular Catheter (central or PICC line)** | **Chest Tube** | **Surgical Drain** | **Diarrhea** | **Rectal Tube** | **NG Tube** |
| --- | --- | --- | --- | --- | --- | --- | --- | --- | --- |
|  |  |  |  |  |  |  |  |  |  |
|  |  |  |  |  |  |  |  |  |  |
|  |  |  |  |  |  |  |  |  |  |
|  |  |  |  |  |  |  |  |  |  |
|  |  |  |  |  |  |  |  |  |  |
|  |  |  |  |  |  |  |  |  |  |
|  |  |  |  |  |  |  |  |  |  |

**Observation Form A**

**HCW SAMPLES DATA COLLECTION SHEET**

| **HCWID:** |  | | | | |  |  |  |
| --- | --- | --- | --- | --- | --- | --- | --- | --- |
| **OBSERVATION DATE:** | | |  |  |  | |  |  |
| **ENTRY TIME:** | |  | XXXXXX |  | **EXIT/END TIME:** | |  |  |

| **PROVIDER TYPE:** | | | | | |
| --- | --- | --- | --- | --- | --- |
|  | Nurse |  | Respiratory Tech |  | MD/Nurse practitioner |
|  | Occupational/Physical Therapy |  | Patient Care Tech (PCT) |  | Environmental Services |
|  | Other (specify): | | | |  |

| **ENVIRONMENTAL CONTACT** | |  | **PATIENT CONTACT** | |
| --- | --- | --- | --- | --- |
|  | Sink |  |  | Physical exam |
|  | Bed Rail |  |  | Wound dressing |
|  | Bedding |  |  | Bathing/hygiene |
|  | Bedside Table |  |  | Catheter/drain |
|  | Vital Sign Monitor |  |  | ETT/trach |
|  | Supply Cart |  |  | Vital signs |
|  | Lift |  |  | Giving meds (oral) |
|  | IV Pump |  |  | IV tubing/IV meds |
|  | Ventilator |  |  | Transfer in/out of bed |
|  | Curtain |  |  | Blood draw |
|  | Trash |  |  | Glucose monitoring |
|  | Computer |  |  | Rectal tube/bag |
|  | Barcode scanner |  |  | Suctioning |
|  | Call button/remote |  |  |  |
|  | Other (specify below) |  |  | Other (specify below) |
|  | |  |  | |

**Patient Quantitative Cultures**

**STUDY ID**: ______________________

If patient REFUSED all, check here:

|  | **Yes** | **No** | **If yes, date**  **obtained** |
| --- | --- | --- | --- |
| Skin 1 (arm) |  |  |  |
| Skin 2 (chest) |  |  |  |
| Perianal |  |  |  |
| Stool (CRE) or Nose (MRSA) |  |  |  |
